# Multi-Organ Gland Segmentation Using Deep Learning

**DOI:** 10.3389/fmed.2019.00173

**Published:** 2019-08-05

**Authors:** Thomas Binder, El Mehdi Tantaoui, Pushpak Pati, Raúl Catena, Ago Set-Aghayan, Maria Gabrani

**Affiliations:** ^1^IBM Watson Health Imaging, Paris, France; ^2^Department of Advanced Analytics, IBM GBS, Paris, France; ^3^IBM Zurich Research, Zurich, Switzerland; ^4^Watson Department, IBM Interactive, Paris, France

**Keywords:** histopathology, segmentation, computer aided diagnosis, stroma, gland, multi-organ, cancer diagnosis

## Abstract

Clinical morphological analysis of histopathology samples is an effective method in cancer diagnosis. Computational pathology methods can be employed to automate this analysis, providing improved objectivity and scalability. More specifically, computational techniques can be used in segmenting glands, which is an essential factor in cancer diagnosis. Automatic delineation of glands is a challenging task considering a large variability in glandular morphology across tissues and pathological subtypes. A deep learning based gland segmentation method can be developed to address the above task, but it requires a large number of accurate gland annotations from several tissue slides. Such a large dataset need to be generated manually by experienced pathologists, which is laborious, time-consuming, expensive, and suffers from the subjectivity of the annotator. So far, deep learning techniques have produced promising results on a few organ-specific gland segmentation tasks, however, the demand for organ-specific gland annotations hinder the extensibility of these techniques to other organs. This work investigates the idea of cross-domain (-organ type) approximation that aims at reducing the need for organ-specific annotations. Unlike parenchyma, the stromal component of tissues, that lies between the glands, is more consistent across several organs. It is hypothesized that an automatic method, that can precisely segment the stroma, would pave the way for a cross-organ gland segmentation. Two proposed Dense-U-Nets are trained on H&E strained colon adenocarcinoma samples focusing on the gland and stroma segmentation. The trained networks are evaluated on two independent datasets, they are, a H&E stained colon adenocarcinoma dataset and a H&E stained breast invasive cancer dataset. The trained network targeting the stroma segmentation performs similar to the network targeting the gland segmentation on the colon dataset. Whereas, the former approach performs significantly better compared to the latter approach on the breast dataset, showcasing the higher generalization capacity of the stroma segmentation approach. The networks are evaluated using Dice coefficient and Hausdorff distance computed between the ground truth gland masks and the predicted gland masks. The conducted experiments validate the efficacy of the proposed stoma segmentation approach toward multi-organ gland segmentation.

## 1. Introduction

Recent developments in computational pathology have enabled a transformation in the field where most of the workflow of the pathology routine has been digitized. A key factor has been the development of cost and time efficiency of whole slide imaging (WSI) scanners as successors of microscope combined with cameras. This process is analogous to the digitization of radiology images. Similar trends have been occurring in other biomedical fields, such as genome analysis, metabolism, proteomics, etc. All of which builds toward the grand vision of computer-assisted precision medicine. Currently, the amount of data available is exceptionally large and far exceeds the rate at which it can be analyzed efficiently. Namely, in pathology, dozens of biopsy samples may need to be collected from patients to characterize a tumor, each leading to gigapixel-sized images. Hence, it is not practical for pathologists and researchers to analyze all of them through visual examination of the specimens.

Therefore, efficient computer aided diagnosis (CAD) in the domain of histopathology to support and improve the decision-making process of experts is necessitated. CAD and image analysis have been developed to assist pathologists and clinicians in cancer diagnosis, prognosis, and treatment recommendation. These systems aim at significantly reducing the labor and subjectivity of traditional manual interventions. The manual analysis of histology tissues remains the primary standard method for cancer diagnosis and depends heavily on the expertise and experience of histopathologists. Compared to other diagnostic technologies, tissue analysis is more invasive, but in most cases provides a better insight on the potential disease and health of the patient.

The process of cancer grading relies almost exclusively on the morphology of the tissues. In clinical practice, One of the important factors toward tissue analysis is the segmentation of glands, which is among the primary criteria to assess the cancer and plan the treatment for individual patient (1, 2). Manual segmentation of glands is time-consuming, laborious, furthermore it remains difficult to reduce variability among experts. Experts currently need to individually highlight all the different glands in a slice. Achieving acceptable levels of reproducibility and robustness remains as an important challenge in the pathology practice (3, 4). Since the success of a team using deep learning at ImageNet Large Scale Visual Recognition Competition (ILSVRC) 2012 (5) an increasing number of the image recognition techniques in CAD development are leveraging the best of deep learning technologies. Similar adoptions have been made in digital pathology. However, the development of deep learning solutions in digital pathology suffer from the unavailability of large annotated training sets, which is essential for the success of deep learning techniques. Several imposing challenges need to be addressed for the development of effective computer-based histology analysis systems:

- Systems need to analyze a large diversity of pathological tissues with any complexity.
- Ability to deal with low amounts of labeled images.
- Address the staining variation and staining artifacts in digital slides, which may appear due to variability in the staining reagents, thickness of tissue sections, staining conditions, scanner models etc.

Developing deep learning techniques toward accurate gland segmentation will enable computer-assisted grading systems to improve the reproducibility and reliability in cancer grading. Gland instance segmentation has been intensively studied over the last few years. Several methods have been investigated and explored such as morphology-based methods (6–9) and graph-based methods (10, 11). With the advent of computer vision and semantic segmentation, it is now possible to design fully convolutional neural networks (FCN) that are able to segment different objects in an image (12, 13). Studies have already shown that some networks are performing well on gland segmentation with Dice scores above 0.85 (12–16). Namely, during the 2015 MICCAI Gland Segmentation (GlaS) Challenge (17), a challenge dedicated to increasing the researchers' interest in gland segmentation, the DCAN (13) displayed the best results on a public single organ dataset of 165 H&E stained colon adenocarcinoma histopathology slides, released for this challenge, using transfer learning and contour computation to separate glands precisely. In addition, a framework proposed by Xu et al. (18) fused complex multi-channel regional and boundary patterns with side supervision for enhanced performances. More recently the MILD-net (14) exhibited the current state-of-the-art results on the same dataset, leveraging U-net architecture (16), contour computation and a pyramidal block aimed at extracting multi-scale features without information loss. Even though these networks show impressive capabilities in term of gland segmentation they rely on single-organ training sets. Glands, or more generally speaking parenchyma tissue, is very different from one organ to the other. It is therefore challenging to design networks able to segment parenchyma from different organs. On the other hand, the stromal component of tissue around the glands is similar in across different organs. This work investigates the interest of focusing on connective tissues, i.e., the stroma, to compute the morphology of glands and support histopathologists in their workflow.

This paper proposes the following approach to address the aforementioned task. It begins with minimizing the staining variability among the H&E images acquired from various sources. Subsequently, deep learning based Dense-U-Net architecture is proposed to segment the glandular structures in the images via two approaches; first, aiming to directly segment the glands, and second, aiming to segment the stroma and then producing the glands from the segmented stroma. Two segmentation networks are trained, following the two approaches, on colon adenocarcinoma H&E images. The predicted gland segmentations are post-processed to improve the segmentation performance. Finally, the trained models are evaluated on two hold-out test datasets from colon adenocarcinoma and breast invasive cancer.

## 2. Dataset Description

**Figure 1:**
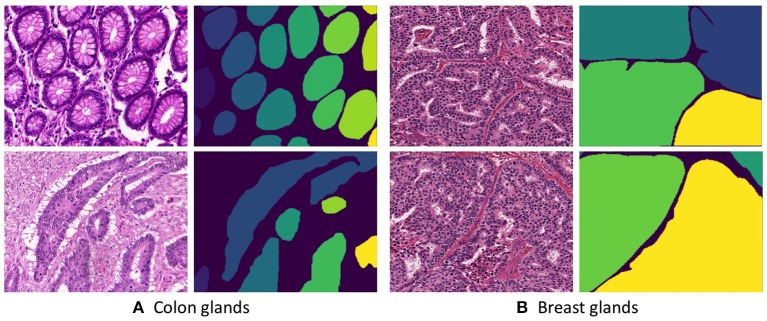
Histopathological slices of (**A** Colon and **B** Breast) tissues and their respective annotated gland segmentation masks.

Two Hematoxylin and Eosin (H&E) stained datasets were used for training and evaluating our proposed methodology. The first dataset contains 165 images derived from 16 H&E stained histological sections of stage T3 or T4 colorectal adenocarcinoma. The T in TNM cancer grading usually refers to the spreading of the primary tumor. Thus, a higher T number implies large tumors that have grown into nearby tissues. In the case of colorectal cancer, the stage T3 implies the tumor has grown into the outer lining of the bowel wall and the stage T4 implies the tumor that has grown through the outer lining of the bowel wall. Notably, the cancer stage is different from the cancer histologic grade as the later indicates the aggressiveness of the tumor (17). The N and M of the TNM grading system describe the state of nearby lymph Nodes and distant Metastasis. Each section belongs to a different patient and sections were processed in the laboratory on different occasions, resulting in high inter-subject variability in both stain distribution and tissue architecture. The images are obtained from 20x magnification of whole-slide images that are digitized by scanning histological sections using a Zeiss MIRAX MIDI Slide Scanner with a pixel resolution of 0.465 μm. The colon dataset is acquired from Gland segmentation challenge (GlaS) 2015 (17). The challenge aimed at precisely segmenting glands in H&E stained slides using image processing techniques. For creating the dataset, 52 visual fields from both malignant and benign areas across the entire set of the whole-slide images were selected to cover as wide a variety of tissue architectures. Each of the visual fields was annotated as benign or malignant by an expert pathologist and the visual fields were further separated into smaller non-overlapping images. Subsequently, the pathologist delineated the boundaries of individual glands in each image. Manual annotations of gland morphologies are used as ground truth for the automatic segmentation. Figure 1A presents a few example images from the colon dataset.

The second dataset is acquired from H&E stained invasive breast cancer WSIs from The Cancer Genome Atlas that are available for the 2016 MICCAI TUPAC challenge (19). The whole-slide images are stored in the Aperio. svs file format as multi-resolution pyramid structures. The files contain multiple downsampled versions of the original image. Each image in the pyramid is stored as a series of tiles, to facilitate rapid retrieval of subregions of the image. The dataset consists of 25 images derived from 25 WSIs across different proliferation scores. An expert histologist annotated the individual gland boundaries on these images. Figure 1B presents a few example images from the breast dataset.

## 3. Methods

The aim is to segment glands in H&E stained tissue images across multiple organs. For this purpose, we propose to build a generic gland segmentation methodology that utilizes gland annotations from only one organ. In this work, we propose to train a deep segmentation network using a gland-annotated colorectal adenocarcinoma dataset and use the trained network for segmenting glands in breast invasive cancer dataset. First, we pre-process the images to get rid of the variation in staining appearance. Second, two approaches are proposed to segment the glands, (a) Gland-approach and (b) Stroma-approach. The same network architecture is used in both the approaches. The Gland-approach is trained with ground truth gland annotations, whereas the Stroma-approach is trained with ground truth stroma annotations, that are derived from the original gland annotations. Third, the trained networks are used to produce gland segmentation masks. The produced gland masks are post-processed using Conditional Random Fields, a class of statistical modeling method. This method uses the predicted labels for the neighboring pixels as context to refine the prediction map. Finally, very small connected component objects are removed and holes are filled in remaining connected components. The individual networks are evaluated on hold-out test datasets from multiple organs.

### 3.1. Pre-processing

**Figure 2:**
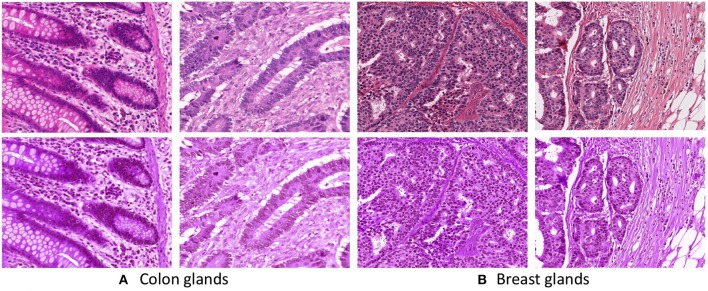
First row presents unnormalized (**A** Colon and **B** Breast) images. Second row presents stain normalized (**A** Colon and **B** Breast) images.

The images in the datasets are acquired from multiple sources and they are captured under different staining conditions. The images possess various staining appearances due to the differences in raw materials, manufacturing techniques of stain vendors, staining protocols of labs, and color responses of digital scanners. To reduce the staining appearance variability, we employ the staining normalization method proposed by Vahadane et al. (20). The method begins with decomposing the images in an unsupervised manner into sparse and non-negative stain density maps. Subsequently, for each image, the method combines its respective stain density map with stain color basis of a pathologist-preferred target image, which results in altering the color of the image while preserving its structure described by the map. Figure 2 presents some examples of the unnormalized and normalized images across all the datasets.

**Figure 3:**
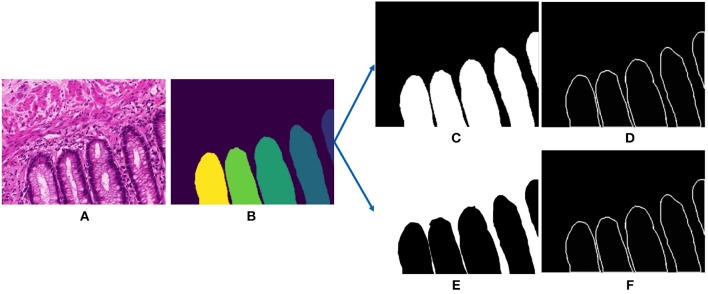
Input and outputs of our deep gland segmentation network. **(A)** Input stain normalized image, **(B)** annotated gland ground truth, **(C)** binarized gland mask, **(D)** binarize gland contour mask, **(E)** binarized stroma mask, and **(F)** binarized stroma contour mask.

Subsequently, we extract the ground truths for training the deep segmentation network. The networks follow a single-input-multiple-output structure, that requires two ground truths for every input image, they are the gland mask and the contour mask. The contour mask is used to delineate the close-by glands. For the Gland-approach, we use the annotated gland masks. The contours were computed from a graph based method consisting in saving the optimal amount of points on the boundary of each glandular object. For the Stroma-approach, we threshold the gray-scale input images to remove the white pixels and then we do pixel-wise intersection with the inverted gland annotations to produce the stroma masks, more details are provided in the next section. For the stroma contour masks, we follow the same approach as the gland contour masks. Figure 3 displays the gland mask, gland-contour mask, stroma mask and stroma-contour mask extracted from an input image and its corresponding gland annotation.

Following this, the training colorectal images and their corresponding ground truth masks are resized to 480×480 pixels. Pixel-wise mean and standard deviation are computed across all the training images. While training, randomly selected images and their corresponding ground truth masks are augmented simultaneously using various on-the-fly augmentation techniques, namely flipping, rotation, elastic transformation, perspective transformation, Gaussian Noise. The augmented images are pixel-wise normalized using the previously computed mean and standard deviation. Due to the diversity of gland morphologies across the training images, perspective transformation results in realistic augmented images.

### 3.2. Deep Architectures

**Figure 4:**
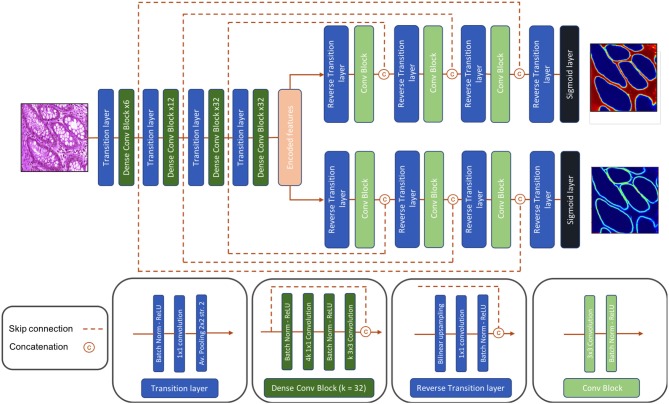
Architecture of our proposed Dense-U-Net segmentation network.

Dense-U-Net, a single-input-multiple-output deep segmentation network architecture, was employed as presented in Figure 4 for both the Gland-approach and the Stroma-approach. The network follows the U-Net (16) type architecture considering its popularity in image segmentation tasks. Unlike U-Net, our network uses asymmetric encoder and decoder. The encoder is designed to automatically and adaptively learn spatial hierarchies of features from low to high level patterns coded within the image. The encoder uses transition layer and dense-convolution blocks consecutively to extract the compressed encoded feature representation. The transition layer down-samples the spatial dimensions of its input by using average-pooling with stride 2, thereby increasing the spatial field-of-view as the network grows deeper. The dense-convolution blocks from DenseNet (21) are used to strengthen feature propagation, encourage feature reuse and substantially reduce the number of parameters as compared to deep residual networks (22). Within a dense-convolution block, direct connections from any layer to any subsequent layers are introduced to ease the information sharing process. This process has two direct consequences. Firstly, the feature-maps learned by any of the DenseNet layers can be accessed by any layer at training time which encourages feature reuse throughout the network and leads to more compact models. Secondly, as individual layers receive additional supervision from shorter connections, dense architectures perform a sort of deep supervision that leads to smoother gradient descent and higher accuracy while retaining a low computational cost. For our network, we use a Dense-169 architecture with [6, 12, 32, 32] numbers of building blocks for the four dense layers with a growth rate of 32.

The decoder is composed of consecutive reverse-transition layers and convolution blocks. The reverse-transition layer up-samples the spatial dimensions of its input using bilinear upsampling. Several reverse-transition layers are included in the decoder to up-sample the encoded features back to the original input image shape. At equal spatial resolution, the output feature maps from a dense-convolution block, from encoder side, is concatenated with the output feature maps from a reverse-transition layer, from decoder side. The concatenated feature maps passes through a convolution operation. The skip connection between the encoder and the decoder side allows for feature reuse and information flow. The architecture has two decoders, one to predict the relevant gland locations, and a second to predicts the gland contours. Thus, the decoders output a gland probability map and a contour probability map. The proposed Dense-U-Net architecture follows an end-to-end training process. The network is trained to jointly optimize the prediction of gland locations and gland contours.

### 3.3. Post-processing

In the Gland-approach, the output gland probability map and the output contour probability map are combined to produce an intermediate gland segmentation mask. Both the output probability maps are thresholded using two preset threshold values to identify the gland pixels and the contour pixels, resulting two binarized masks. The binarized contour mask undergoes two concurrent dilations. Then the dilated contour mask is multiplied with the binarized gland mask to delineate the overlapping glands, thereby accurately identifying the individual glands. Afterwards, the gland mask is dilated by the same factor to recover the boundary information removed by the dilated contour mask. In the Stroma-approach, the output stroma probability map is thresholded by a preset threshold value and the binarized stroma mask is inverted. The inverted stroma mask indicates the gland components similar to the binarized gland mask from above, thus the inverted stroma mask is processed in the similar way as described above.

To produce a better quality gland segmentation, the gland mask was processed through three post-processing steps. (i) Small connected components with an area lesser than a threshold are removed. (ii) Holes inside remaining connected components are filled. (iii) Finally, a conditional random field (CRF) layer, (23, 24) a probabilistic graphical model used in semantic segmentation, is implemented to sharpen and smooth the predictions.

### 3.4. Training Details

The deep segmentation network architecture and the training procedure is identical in both the Gland-approach and the Stroma-approach. The colon gland dataset consists of 165 images from the GlaS challenge. This dataset is split into a training set of 135 images and a test set of 30 images retaining a balance between the number of benign and malignant cases in both training and testing. The training dataset is further split as 80 and 20% into training set and validation set, respectively. The Gland-approach is provided with the ground truth gland masks and contour masks, whereas the Stroma-approach is provided with the ground truth stroma masks and stroma contour masks. Randomly selected images from the training set and their corresponding ground truth masks are augmented on-the-fly at training time. The augmentation increases the number of training samples and enhances the generalization potential of the network.

The dense convolutional blocks and transitional layers in the encoder of the Dense-U-Net is initialized by the DenseNet-169 weights, pre-trained on the ImageNet challenge. The bilinear up-sampling layers and convolution filters in the decoder are initialized by He-normal initialization. The loss constitutes of three terms; a binary cross-entropy gland loss, a binary cross-entropy contour loss and L2 regularization loss, as given in Equation (1). In the Stroma-approach, the binary cross-entropy losses in 1 are computed with respect to ground truth stroma and ground truth stroma contour. The augmented ground truth gland masks and ground truth contours are compared with the sigmoid layer outputs of the network to compute the binary cross-entropy losses. The gland loss and the contour loss are weighted using a hyperparameter α, which is optimized through cross-validation and is set to 0.5. The loss function is optimized using Adam, an adaptive learning rate optimization technique, and backpropagation. We train our Dense-U-Net with Adam parameter update and a learning rate of 1*e*−3. The learning rate is divided by 2 when the validation loss does not improve for 5 epochs. The first and second momentum decay rates are set to 0.90 and 0.99, respectively. The batch sizes is set to 4 and training is regularized by L2 weight decay of λ = 1*e*−4. The networks are trained for 40 epochs and each epoch consists of 200 mini-batches. Validation loss is considered as the metric for selecting the final trained model. Our implementations are derived from Python based Keras framework and are trained on NVIDIA Tesla P100 GPU machine.

(1) $$\begin{array}{r} L_{total}=\alpha \;L_{gland}+\left(1-\alpha \right)L_{contour}+\lambda \;{∥\;w\;∥}^{2} \end{array}$$

## 4. Evaluation Metric

The performance of the segmentation algorithm is evaluated based on two criteria: (1) volume-based accuracy of the segmentation of glands; and (2) boundary-based similarity between glands and their corresponding segmentation. The volume-based metric for segmentation accuracy is calculated using the label that the algorithm assigns to each pixel, and the boundary-based metric uses the position assigned by the algorithm to the boundary of each gland.

The volume-based accuracy is computed using the Dice coefficient. It measures the segmentation accuracy at the pixel level. It is a statistical gauge of the similarity between two sets of samples. Given *G*, a set of pixels belonging to a ground truth object, and *S*, a set of pixels belonging to a segmented object, the Dice coefficient is defined as in Equation (2), where |.| denotes set cardinality. It ranges from 0 (no overlap between *G* and *S*) to 1 (perfect overlap between *G* and *S*).

(2) $$\begin{array}{r} Dice\left(G,S\right)=\frac{2|G\cap S|}{\left|G|+|S\right|} \end{array}$$

The boundary-based segmentation accuracy between the segmented objects in *S* and the ground truth objects in *G* are measured using the Hausdorff distance. It measures how far two subsets of a metric space are from each other. Considering the two sets *S* and *G*, the Hausdorff distance is defined as in Equation (3), where *d*(*x, y*) denotes the distance between pixels *x* ∈ *G* and *y* ∈ *S*. In this work, we use the Euclidean distance. The Hausdorff distance represents the longest distance from *S* (respectively *G*) to its closest point in *G* (respectively *S*). It is the most extreme value from all distances between the pairs of nearest pixels on the boundaries of *S* and *G*. Two sets display a low Hausdorff distance if each point of either set is close to another of the second set, i.e., Hausdorff distance = 0 iff *S* = *G*.

(3) $$\begin{array}{r} d_{H}\left(S,G\right)=max\left[\underset{x\in G}{\sup}\underset{y\in S}{\inf}d\left(x,y\right),\underset{y\in S}{\sup}\underset{x\in G}{\inf}d\left(x,y\right)\right] \end{array}$$

In this work, the ground truth binary gland masks are compared with the predicted gland segmentation masks via Dice coefficient and directed Hausdorff distance. A higher Dice coefficient and a lower Hausdorff distance indicate the efficacy of the gland segmentation method.

## 5. Results

Glands are the most important structures for establishing the malignant tumor's TNM classification, thus stromal tissue segmentation has never been thoroughly investigated. For this study, two gland segmentation approaches are explored, namely Gland-approach and Stroma-approach. The Stroma-approach has two important consequences. First, the pathologists are more interested in gland morphologies than the stroma localization, consequently it is important to come up with a relevant and robust algorithm to compute gland masks from stroma masks and vice versa. Indeed, the stroma mask is not an exact match to the complementary mask of the glands'. Namely, there are several white regions that are neither gland nor stroma. Second, there is no expert annotated data for stroma masks. Without stroma masks at disposal, it is necessary to automatically compute stoma masks from gland masks. We use an inversion algorithm based on three distinct types of tissue: parenchyma, white areas and stromal tissue. Stroma masks are computed and then validated with an expert histologist to assess their correctness. It ensures access to a fair amount of annotated data and the ability to compute the glands location from the stroma mask. Both the approaches are trained using gland-annotated colon adenocarcinoma dataset. For the multi-organ gland segmentation evaluation, the networks are evaluated on two independent datasets from colon adenocarcinoma and breast invasive cancer. The results are presented in the following sections.

### 5.1. Segmentation Results on the Colon Dataset

**Figure 5:**
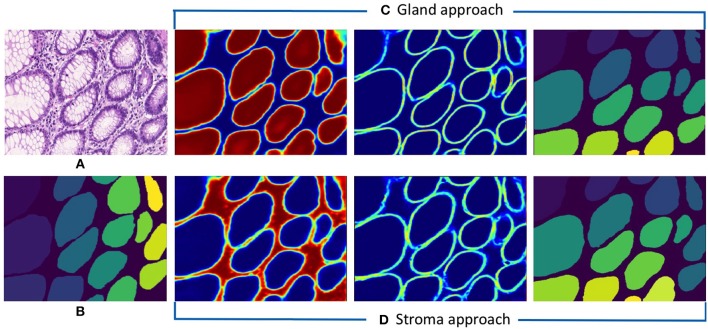
Segmentation results for colon adenocarcinoma dataset. **(A)** Original colon image, **(B)** annotated ground truth gland mask, **(C)** predicted gland probability mask, gland contour mask, and final gland segmentation results with Gland-approach, and **(D)** predicted stroma probability mask, stroma contour mask, and final gland segmentation results with Stroma-approach.

**Figure 6:**
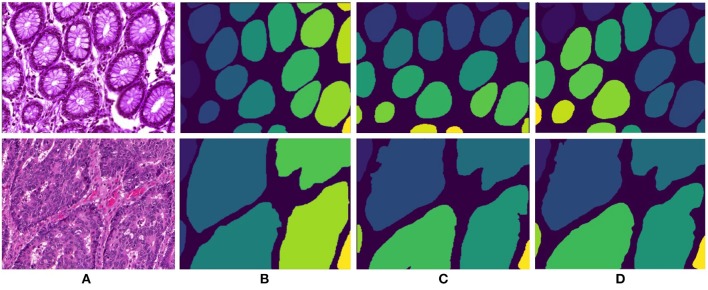
Segmentation results for colon adenocarcinoma dataset. First row and second row presents results for benign and malignant colon images respectively. **(A)** Original colon image, **(B)** annotated ground truth gland mask, **(C)** final gland segmentation results with Gland-approach, and **(D)** final gland segmentation results with Stroma-approach.

Following the aforementioned workflow, Dense-U-Nets are trained via the Gland-approach and the Stroma-approach using the colon GlaS dataset. Then, both approaches are tested on colon adenocarcinoma slides to assess the performance on a single organ. Figure 5 displays a visual sample of segmentation results from an original colon H&E stained slide. Qualitative evaluation indicate similar performance for both the approaches. It is further supported by quantitative evaluation via the Dice coefficient and the Hausdorff distance criteria presented in Table 1. Both approaches perform similarly in terms of the Dice coefficient. However, the Stroma-approach performs better in terms of the boundary segmentation, as indicated by the Hausdorff distance. The post-processing seemed not to improve the segmentation results for the Gland-approach, whereas it slightly improves the results for the Stroma-approach. Since, the stroma masks are computed automatically it is difficult to exactly reason the performance gap between the two approaches. Figure 6 presents two more segmentation results for benign and malignant colon glands using Gland- and Stroma-approach.

**Table 1:** Dice coefficient and Directed Hausdorff distance table for different experiments.

	**Dice coefficient**		**Hausdorff distance**	
	**Gland-app**.	**Stroma-app**.	**Gland-app**.	**Stroma-app**.
Colon: before post-processing	0.93	0.92	10.8	10.5
Breast: before post-processing	0.76	0.84	13.9	13.8
Colon: after post-processing	0.92	**0.93**	11.0	**9.7**
Breast: after post-processing	0.78	**0.87**	13.6	**11.2**

*Bold values indicates the best results for the Stroma Approach*.

### 5.2. Segmentation Results on the Breast Dataset

**Figure 7:**
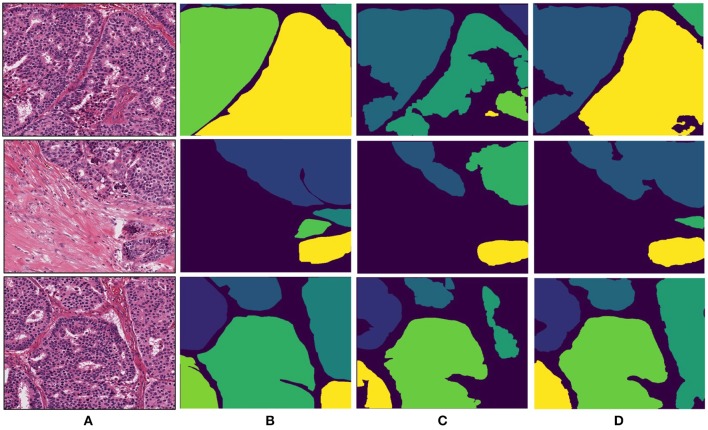
Segmentation results for breast invasive cancer dataset. **(A)** Original breast image, **(B)** annotated ground truth gland mask, **(C)** final gland segmentation results with Gland-approach, and **(D)** final gland segmentation results with Stroma-approach.

The breast dataset images have a higher spatial dimension than the previous colon images, i.e., 2000×2000 pixels. The best performing models of the Gland-approach and Stroma-approach, trained on the GlaS dataset, are tested on the breast dataset to assess their generalizing ability toward gland segmentation in a new organ, that is unknown to the trained models. We evaluate the approaches on the acquired breast dataset with 25 annotated images. First, the breast H&E stained images are mapped to the same color distribution as of the GlaS dataset (Figure 2). The staining normalization step is of the utmost importance as it minimizes the staining variability between both the domains. Second, the pre-trained Dense-U-Nets on colon dataset are employed to predict the gland segmentation masks in the breast dataset. Three breast samples, their corresponding ground truth gland annotations, the gland predictions via the Gland-approach and Stroma-approach are presented in Figure 7. Qualitative evaluation via visual inspection indicate that the Stroma-approach is able to identify the individual glands and is more consistent with the ground truth annotations compared to the Gland-approach. The Gland-approach fails to generalize to new organs owing to the high variation in the gland morphologies across organs. However, the Stroma-approach display a reasonable performance in generalizing to new organs, as it targets to delineate the more consistent stroma across organs. The visual intuitions are further supported by the computed Dice coefficients and Hausdorff distance metrics in Table 1.

## 6. Discussion

Analysis of glandular morphology in H&E stained histopathology slides is among the primary factors in cancer staging and thereby selecting the treatment procedure. However, visual assessment is tedious and time consuming as pathologists are required to manually examine each specimen to perform an accurate diagnosis. Moreover, due to its complex behavior, histopathological diagnosis suffers from inter- and intra-expert variability. Therefore, designing an automated system enabling precise assessment of the gland morphologies amongst not only one but multiple organs of the body will be a considerable breakthrough in the pathologists' routines. This would pave the way for a quicker and bias-free method of cancer diagnosis as well as supporting the clinicians in prescribing the treatment procedure. To improve the gland segmentation and extending to a multi-organ setting, this paper proposes a stroma segmentation approach, and subsequently presents a stroma to gland delineation method. The proposed methodology is developed using colon adenocarcinoma tissue samples and extends to cross-organ gland segmentation. The significance of the methodology is established by evaluating it on samples from colon dataset (same organ) and samples from breast invasive cancer (different organ). The method is compared against a direct gland segmentation approach, which is developed and evaluated on the same datasets as of the first approach.

Qualitative evaluation via visual inspection and quantitative evaluation via Dice coefficient and Hausdorff distance on datasets from two organs support the generalizing ability of the Stroma-approach compared to the Gland-approach. The model trained via Gland-approach achieves Dice coefficients of 0.92 and 0.78 on the colon and breast test datasets respectively, whereas the Stroma-approach is produces Dice coefficients of 0.93 and 0.87 on the same test datasets. The Dice coefficients of the Gland-approach and Stroma-approach drop by 15.22 and 6.45%, respectively on changing the test dataset from colon to breast. The Hausdorff distances for the Gland-approach and Stroma-approach increases on changing the evaluation from colon to breast dataset. However, the Stroma-approach has a better Hausdorff distance compared to the Gland-approach. Visual analysis of Figure 7 demonstrates that on the breast dataset, unknown to the trained networks, the Stroma-approach is able to delineate the glands more precisely compared to the Gland-approach. The edgy segmentation of the Stroma-approach occurs due to automatically computed ground truth stroma masks. The stroma masks are not simple inversions of gland masks and do not produce the same accuracy after automated computation. As the ground truth for Stroma-approach do not benefit from the precise knowledge of experts it is harder for the network to accurately segment the boundaries between glands and stroma. This implies that a deep architecture trained on precise stroma masks would exhibit better performances on multi-organ segmentation. This paper notes that more efforts and time should be put into this area to leverage the best from stroma segmentation, however this preliminary study conveys very promising results for cancer diagnosis and grading on a multi-organ scale. The importance and relevance of the stromal tissue for accurate gland morphology assessment is confirmed to design a cross-organ automated annotation tool using deep learning.

This work has the ambition to serve as a proof of concept that introduces a new cross-organ gland segmentation strategy, leveraging the consistency of stromal tissue across different organs. The ability of neural network based on convolutional layers to isolate and locate this tissue would greatly benefit any specialist aiming to measure gland morphologies within the cancer grading workflow. This approach can further serve higher purposes in the future in the problematic of classification between benign and malignant tumors after segmentation. Namely, a second deep architecture could benefit from the measurements of the first architecture to establish a classification suggestion. This represents an incremental advance in the pursuit of supporting the treatment suggestion decision-making of clinicians. This first approach for a cross domain application goes into the right direction.

## Data Availability

The raw data supporting the conclusions of this manuscript will be made available by the authors, without undue reservation, to any qualified researcher.

## Author Contributions

PP and RC conceived the idea for this study. TB worked on the end-to-end implementation of the study. ET and PP technically supervised TB throughout the implementation process. RC developed the software for image annotation, annotated the breast dataset, and assessed the experimental results. AS-A as subject matter expert provided relevant insights on the business and clinical impact of the research work for histopathologists. Redaction of the paper was handled by TB, ET, and PP, supported by AS-A and RC. MG the head of the computation pathology team from IBM Research Zurich, provided part of the funding for the research and managed the collaborative work with GBS France.

### Conflict of Interest Statement

Author TB was employed by company IBM Watson IMaging, authors PP, RC, and MG were employed by IBM Zurich Research and authors ET and AS-A were employed by IBM GBS. All the authors declare that the research was conducted in the absence of any commercial or financial relationships that could be construed as a potential conflict of interest.
